# Socioeconomic, Geospatial, and Geopolitical Disparities in Access to Health Care in the US 2011–2015

**DOI:** 10.3390/ijerph14060573

**Published:** 2017-05-29

**Authors:** Samuel D. Towne

**Affiliations:** Department of Health Promotion and Community Health Sciences, School of Public Health, Texas A&M University, College Station, TX 77846, USA; towne@sph.tamhsc.edu

**Keywords:** health disparities, environmental and social predictors, place-based disparities, access to care

## Abstract

Individuals forgoing needed medical care due to barriers associated with cost are at risk of missing needed care that may be necessary for the prevention or maintenance of a chronic condition among other things. Thus, continued monitoring of factors associated with forgone medical care, especially among vulnerable populations, is critical. National survey data (2011–2015) for non-institutionalized adults residing in the USA were utilized to assess forgone medical care, defined as not seeking medical care when the individual thought it was necessary because of cost in the past 12 months. Logistic regression was used to predict forgone medical care vs. sought medical care. Racial/ethnic minority working-age adults, those with lower incomes, those with lower educations, those residing in the South, and those residing in states that failed to participate in Medicaid Expansion in 2014 were more likely (*p* < 0.01) to forgo medical care due to cost in the past year. Policy makers seeking to reduce barriers to forgone medical care can use this information to tailor their efforts (e.g., mechanisms targeted to bridge gaps in access to care) to those most at-risk and to consider state-level policy decisions that may impact access to care.

## 1. Introduction

Gaps in access to medical care must be addressed in a timely and efficient manner, with an understanding of risk factors for those most at-risk. The continued monitoring of factors influencing access and utilization of needed medical care, especially among vulnerable populations, is critical in ameliorating health disparities. When individuals decide to forgo needed medical care, this can lead to potentially preventable gaps in accessing preventative services [1,2]. This is a timely issue, especially as some geopolitical decisions regarding health care access can be made at the state level and may affect at-risk populations, having health-related implications for millions in the US [3].

Socioeconomic factors such as income may influence whether an individual seeks medical care when they believe it necessary [4,5]. Further, differences in forgone medical care have been shown throughout times of economic downturns with particular gaps in access to medical care facing minority adults [5]. These gaps in access to needed care have been shown to be different across social determinants of health [6,7,8].

### 1.1. Structural and Social Determinants of Health

The World Health Organization’s Framework for Action on the Social Determinants of Health [6] postulates that health and health-related outcomes can be influenced by the structural and social determinants of health and health-related outcomes. Specifically, race/ethnicity, sex, education, and individual-level socioeconomic factors (e.g., income) serve as social determinants of health in this framework [6]. In addition to these individual-level factors, the structural determinants of health may include socioeconomic measures (e.g., macroeconomic policies) of larger geographic areas such as countries or states and public policies, including but not limited to health policies [6]. Area-level characteristics that may impact access to medical care may include region [9], state socioeconomic variables based on income/poverty [5,9], and geopolitical characteristics, including Medicaid policies, which are based on research into early evaluations of Medicaid expansion that included a smaller geospatial scope (i.e., three US states with expansion relative to comparisons of non-expansion states) [3]. Major disparities related to access to medical care have been found in the US across several individual-level outcomes (e.g., race, income) [4]. Further, geospatial correlates of forgone medical care have been shown in earlier research where forgone medical care was found to be more likely in certain geographic regions (e.g., the south) [10].

Large national datasets (e.g., the Behavioral Risk Factor Surveillance System (BRFSS)) have been utilized to show state-specific correlates (e.g., weak health care infrastructure) of forgone medical care [10]. State-specific public policies (e.g., Medicaid coverage decisions) have also been shown to impact forgone medical care and mortality in a limited selection of states in the US [3].

### 1.2. Objective

The Framework for Action on the Social Determinants of Health [6] was used to inform the current study’s aims: (1) identify the individual-level factors associated with forgone medical care after the Great Recession and (2) identify the geospatial and geopolitical factors influencing forgone medical care. This study adds to the literature by providing a timely evaluation, including the most recent national data, of several policy-relevant issues facing vulnerable adults, as related to access to medical care.

## 2. Materials and Methods

### 2.1. Data

The Behavioral Risk Factor Surveillance System (BRFSS) was the primary dataset used in the current analyses. The 2011 (*n* = 506,467), 2012 (*n* = 475,687), 2013 (*n* = 491,773), 2014 (*n* = 464,664), and 2015 (*n* = 441,456) BRFSS public use files were used [11]. The BRFSS is an annual telephone survey of non-institutionalized adults.

### 2.2. Dependent Variables

Forgone medical care was the primary outcome of interest in the current study. The survey item included ‘Was there a time in the past 12 months when you needed to see a doctor but could not because of cost?’ [11]. This was coded as yes vs. no.

### 2.3. Independent Variables

The BRFSS was used to measure all individual-level variables. Income was coded as Don’t know/Not sure/Missing, Less than $15,000, $15,000 to less than $25,000, $25,000 to less than $35,000, $35,000 to less than $50,000, and $50,000 or more. Sex was coded as male or female. Education was coded as Did not graduate High School, Graduated High School, Attended College or Technical School, or Graduated from College or Technical School.

Race and ethnicity was included as non-Hispanic White, non-Hispanic Black or African American, non-Hispanic American Indian or Alaska Native, non-Hispanic Asian, and Hispanic. An ‘other’ category was included, defined as individuals reporting any of the following classifications: Native Hawaiian or Other Pacific Islander, No preferred race, Multiracial but preferred race not asked, Don’t know/Not sure, or Refused. Those adults categorized as ‘other’ in terms of race/ethnicity are reported in the tables but not in the text, given it was not possible to detect a particular race/ethnicity among individuals classified as ‘other’ with the BRFSS.

#### 2.3.1. Neighborhood-Level Independent Variable

Rurality was included to assess the differences across rural and urban areas, given it has been shown to be associated with access to care [12]. This variable was taken from the BRFSS. This included inside a metropolitan statistical area vs. not in a metropolitan statistical area. This was the only variable available to measure rurality included in the BRFSS. The use of this included measure (i.e., in the BRFSS data) of rurality is consistent with previous research highlighting broad rural and urban differences [13], yet it does not allow for a more detailed measure of variation within rural areas (i.e., given they are grouped into a single category of non-metropolitan).

#### 2.3.2. State-Level Independent Variables

State average median household income was used for each year under study. This variable was included to incorporate a measure of socioeconomic differences between states. The US Census Small Area Income and Poverty Estimates data was used to measure state average median household income.

Medicaid Expansion was included to have a state-level measure of geopolitical policy. The states expanding Medicaid as of 1 January 2014 were included in the definition of Medicaid Expansion. The following footnotes were taken directly from the Kaiser Family Foundation website (the source of the data on Medicaid Expansion for the current analyses): ‘Coverage under the Medicaid expansion became effective 1 January 2014 in all states that have adopted the Medicaid expansion except for the following: Michigan (1 January 2014), New Hampshire (15 August 2014), Pennsylvania (1 January 2015), Indiana (1 February 2015), Alaska (1 September 2015), Montana (1 January 2016), and Louisiana (1 July 2016).’ Source: (http://kff.org/health-reform/state-indicator/state-activity-around-expanding-medicaid-under-the-affordable-care-act/?currentTimeframe=0). The classification of states as expanding or not, as included in the presented analyses, is defined as all states expanding as of 1 January 2014 vs. all other states. Given that additional states expanded Medicaid at different time points in addition to those expanding as of 1 January 2014, additional models were constructed.

The following definitions of states that underwent Medicaid Expansion were used: **Definition (1)** 25 states, including Arizona, Arkansas, California, Colorado, Connecticut, Delaware, District of Columbia, Hawaii, Illinois, Iowa, Kentucky, Maryland, Massachusetts, Minnesota, Nevada, New Jersey, New Mexico, New York, North Dakota, Ohio, Oregon, Rhode Island, Vermont, Washington, and West Virginia (these results are presented in the tables in the current study); **Definition (2)** 26 states total, including Michigan (expansion 1 January 2014); **Definition (3)** 27 states total, including Michigan and New Hampshire (15 August 2014); and **Definition (4)** 30 states, including Michigan, New Hampshire, Pennsylvania (1 January 2015), Indiana (1 February 2015), and Alaska (1 September 2015). The definitions are cumulative, wherein Definition 4 includes all states in Definition 1–3. Models by year were run for each of the definitions (1–4). All models indicated that the results for the Medicaid Expansion variable (expanded vs. did not) for 2015 were consistent in terms of significance (*p* < 0.01) and the direction of the odds ratio (OR). Thus, the decision to present only those results for the most restrictive definition (25 states expanding 1 January 2014 vs. all other states) was made. This decision was made because the definition of the outcome of interest (forgone medical care) was based on a time in the past 12 months when an individual did not seek medical care when they thought it was needed due to cost.

### 2.4. Statistical Analyses

Logistic regression was used to model the dichotomous variable, forgone medical care. SAS 9.4 (Cary, NC, USA) was used in all analyses. SAS survey procedures were utilized to incorporate the complex sampling frame of the data. These include ‘proc surveylogistic’ for fully-adjusted analyses. The fully adjusted analyses include income, sex, education, race/ethnicity, rurality (urban or rural), census region (north, south, east, and west), and the state average median household income by year. Medicaid Expansion is added to this model only where indicated in tables. The fully-adjusted analyses were separated into those adults aged 18–64 and those adults aged 65 and older.

### 2.5. Ethics

Approval for this secondary data analysis was gained from the Texas A&M University’s Institutional Review Board. On 6 February 2013, this public use data file was reviewed by the Texas A&M IRB with the intent of making these data files available for public use. Therefore, no further review by the Texas A&M IRB is required for studies involving these public use data files.

## 3. Results

### 3.1. Forgone Medical Care

#### 3.1.1. Working Age Adults (Age 18–64 Years)

Table 1 presents the distribution of outcomes of interest by key characteristics. Overall, nearly 20% of adults between the ages 18 to 64 reported a time when they did not seek health care services because of cost in the past 12 months (forgone medical care). This dropped to 15% in 2015. The rates were highest for adults with the lowest incomes (less than $15,000 annually) and among adults with the lowest education levels (without a high school degree or equivalent). The rates of forgone medical care were also highest for Hispanic adults, followed by Black or African American and American Indian or Alaska Native adults (not including those categorized as ‘other’) at nearly twice the rates of White or Asian adults. Further, individuals in rural areas had higher rates of forgone medical care than those in urban areas. The rates of forgone medical care were highest in the South.

#### 3.1.2. Older Adults (Age 18–64 Years)

Among those 65 years of age and older, the rate of forgone medical care remained at nearly 5% for all years under study. The rates of forgoing medical care were highest among those with the lowest incomes. Similarly, the rates were highest among those with the lowest education. Aside from those adults categorized as ‘other’ for race/ethnicity, American Indian or Alaska Native elders faced the highest rates of forgone medical care in 2011 and 2012, with Hispanic older adults having the highest rates in 2013, 2014, and 2015. The rates of forgone medical care were somewhat similar, albeit with higher rates in rural areas (with less than a percentage point difference). The rates of forgone medical care were highest in the South relative to all other census regions.

#### 3.1.3. Bivariate Analyses

Table 2 presents bivariate analyses of forgone medical care by state Medicaid Expansion in 2014. Odds ratios (OR) predicting forgone medical care were run for comparisons of states participating in Medicaid Expansion or not in January of 2014. The results indicated higher rates of forgone medical care in non-expansion states for each year among those ages 18–64, with the largest difference measured for 2015 (OR 1.37, 99% Confidence Interval or CI 1.30–1.44). No significant variation was measured for those 65 and older.

### 3.2. Multivariable Logistic Regression for Forgone Medical Care

#### 3.2.1. Working Age Adults (Age 18–64 Years)

The factors significantly (*p* < 0.01) and consistently (across all years under study) associated with forgone medical care among adults ages 18–64 included income, sex, education, race/ethnicity, and census region after adjusting for all other variables in the model (see Table 3). Individuals with lower income categories vs. highest incomes (at or above $50,000 annually) were more likely to report forgone medical care (*p* < 0.01) in all years under study. Males were less likely (*p* < 0.01) to report forgone medical care than females. Individuals in all other education categories except the highest (Graduated from College or Technical School) were more likely to report forgone medical care after adjusting for all other variables in the model. Hispanic adults were more likely (*p* < 0.01) to report forgone medical care than White adults in all years under study after adjusting for all other variables in the model. Further, individuals in all other census regions outside of the South were less likely to report forgone medical care in all years under study after adjusting for all other variables in the model.

When running models that included a categorization of whether individuals resided in a state that expanded Medicaid in 2014, many similar patterns were found for income, sex, education, race/ethnicity, and census region after adjusting for all other variables in the model (see Table 4). However, after including a categorization for whether states participated in the Medicaid Expansion, only after the Medicaid Expansion was actually adopted (in 2014 and 2015) were there significant differences reported in the adjusted analyses. The results suggest that individuals in states that did not expand Medicaid were more likely (*p* < 0.01) to report forgone medical care after adjusting for all other terms in the model.

#### 3.2.2. Older Adults (Age 18–64 Years)

The factors significantly (*p* < 0.01) and consistently (across all years under study) associated with forgone medical care among older adults ages 65 and older included income, education, and race/ethnicity after adjusting for all other variables in the model (see Table 5). Individuals with lower income categories vs. the highest income category (at or above $50,000 annually) were less likely to report forgone medical care (*p* < 0.01) in all years under study. Individuals without a high school diploma or equivalent vs. the highest education category (Graduated from College or Technical School) were more likely (*p* < 0.01) to forgo medical care in all years under study. Further, older adults that were classified into a racial or ethnic minority group were more likely than White older adults to report forgone medical care in all years under study after adjusting for all other variables in the model.

When running models that included a categorization of whether individuals resided in a state that expanded Medicaid in 2014, many similar patterns were found for income, sex, education, race/ethnicity, and census region after adjusting for all other variables in the model (see Table 6). However, after adjusting for the Medicaid Expansion in select states, there were significant differences reported in the adjusted analyses in 2012 and 2014. The results suggest that differences were present both before the Medicaid Expansion was enacted (in 2012) and after the initial Medicaid Expansion in 2014 but not in 2015, indicating no consistent (i.e., over time) differences either before or after the Medicaid Expansion in 2014 after adjusting for all other terms in the model.

## 4. Discussion

Understanding factors associated with forgone medical care among both younger (18–64 years) and older adults (age 65 and older) is critical if we are to seek solutions that ameliorate gaps in access to and utilization of medical care. This is especially crucial given the relative disparities found in the current study among potentially vulnerable populations. Our findings that both sociodemographic factors (education, sex, race/ethnicity, age), socioeconomic factors (income), geospatial factors (rurality, census region), and geopolitical factors (state Medicaid Expansion or not) may play a role in whether an individual reports forgone medical care, which helps to inform research, practice, and policy as it relates to linking individuals to necessary medical care. The results of the current study confirm the utility and appropriateness of the Framework for Action on the Social Determinants of Health [6]. This framework has been used in similar analyses assessing forgone medical care throughout the Great Recession [5], with similar findings for individual-related social determinants of health. However, the current study provides the most recent available national estimates of forgone medical care using data that includes a timeline of multiple years post-Medicaid expansion and other mechanisms of the Affordable Care Act (2010).

Clear health disparities were noted among those with lower incomes and lower education levels and those classified as being from a racial or ethnic minority group. This confirms the results of previous studies identifying, among other factors, race/ethnicity, income, and education as key determinants of health and health-related outcomes [4,5,6]. The findings related to health-related disparities among particularly at-risk individuals, including, but not limited to, individuals with low incomes, those with relatively low levels of education, those in rural areas, those in the South, and those from racial or ethnic minority groups, are particularly timely given that these historic disparities continue to persist into and beyond the economic recovery.

Further, the finding that those in the South were more likely to report forgone medical care is also timely and relevant given that several states in the South are currently not planning to expand Medicaid at this time. The findings in fully adjusted analyses indicate that individuals in states expanding Medicaid were less likely to report forgone medical care due to cost, even after adjusting for all other terms in the model. Of critical note, these findings related to Medicaid Expansion are associative and not assumed to be causal given the current cross-sectional analyses. Even so, this is of potential use to policy makers in these states tasked with deciding to participate in Medicaid Expansion in the future. More research must be done that incorporates longitudinal analyses with a specific focus on those most likely to qualify for Medicaid. While the results of the current study related to Medicaid Expansion are crude in nature, do not imply causality, and were not restricted to only those eligible for Medicaid in a given state, they do add additional insight into the relevance of states’ decisions to participate in Medicaid Expansion. Our results are consistent with similar analyses showing that individuals residing in states that did not expand Medicaid were more likely to face gaps in accessing medical care [3].

Given the rapidly aging population in the US [14], our findings related to older adults are also timely and relevant to informing efforts aimed at ensuring adequate access to health care for this potentially vulnerable population. In 2050, it is estimated that older adults will make up nearly 84 million individuals, up from nearly 43 million in 2012 in the US [14]. With over nine in 10 adults aged 65 and older having at least one chronic condition, and nearly three in four having two or more chronic conditions [15], it will be critical to ensure adequate access to care for this particularly at-risk population. Given that older adults are traditionally covered by Medicare, it was not surprising that these individuals had much lower rates of forgone medical care as compared to those individuals ages 18–64 years. However, even among this older age group, clear disparities were noted by income, education, and race/ethnicity. Identifying policies that enable older adults to be financially stable into older age can be crucial, even among a population traditionally served by Medicare.

## 5. Limitations

Several limitations should be considered in the current study. First, multiple cross-sections of data were used in the current study. Thus, causality is not implied. Even so, multiple years of data were incorporated to measure changes over time. The majority of the data used in the current study are based on participant self-reported responses. As such, recall bias may influence the results. While the limitations of surveys are common to health research, the ability to use nationally representative samples for each year under study was a major strength.

The measure of Medicaid Expansion was limited given that there is no distinction between differing approaches or the implementation of Medicaid Expansion by states. Further, the experience of individuals within states that participated in Medicaid Expansion is likely not equivalent given state variation across a number of unmeasured factors (e.g., other measures of geopolitical variation). While Medicaid Expansion was included as a geopolitical measure, we did not restrict the results to only those adults that were recipients of Medicaid or new recipients. This measure used in this study is a crude categorization of geopolitical variation among and between states, with several other policy-relevant differences not taken into account. A major limitation of the data was not being able to determine who would be eligible for Medicaid and who was actually enrolled. Further, the implementation of American Health Benefit Exchanges and Small Business Health Options Program (SHOP) also followed a similar timeline as Medicaid Expansion in the current study as it was implemented starting in 2014. There was no measure of enrollment in exchanges in the current study.

Further, there are several factors influencing forgone medical care that cannot be measured in the current study with the data used, including, but not limited to, economic development of smaller geographic areas (smaller than states), including variations in employment opportunities; a measure for the level of dissemination of information about enrolment or uptake of Medicaid or state health insurance exchanges; individual personal perspectives on Medicaid or state health insurance exchanges in principle or related to perceived stigma; and other individual factors influencing an individual’s likelihood to enroll or not in either Medicaid or state health insurance exchanges. In addition, employment was not added to the model. Employment may be correlated to health through indirect (e.g., economically) and direct (e.g., depression) means [16,17]. It is beyond the scope of this study to measure all possible variables that may influence forgone medical care, especially given that no one dataset encompasses all relevant measures. Thus, the implications of the current analyses, data, and crude measures of geopolitical policies should be taken in light of these and other limitations of the current study.

Further, neighborhood characteristics have been shown to impact access to care [18,19]. For example, the ability to include measures of neighborhood segregation [18,19,20] and rurality [21] has been shown to identify potential gaps in access to health care for potentially at-risk groups. Thus, having multiple characteristics measured at a smaller scale than the state may be necessary when identifying a more complete identification of measures associated with gaps in access to care. These and similar measures are recommended for future research.

### Practice and Policy Implications

This study highlights the need for the continued evaluation of policy-relevant issues affecting vulnerable populations in the US. The timeliness of the data provides the most up-to-date evaluation of several factors that have been associated with forgone medical care for working age and older adults since the Great Recession and the passing of major health care legislation (e.g., the Affordable Care Act (2010)). Tailored policies (e.g., mechanisms targeted to bridge gaps in access to care) that focus on access issues facing those most at-risk are needed. In addition, policy makers may use this study as they consider state-level policy decisions (e.g., Medicaid Expansion, future/pending health care-related policies) that may impact access to care, expecially for the most vulnerable. This study highlights not only indiviual-level factors that may serve as targets for action but also the importance of place-based factors that may serve to guide efforts at a geoplolitical level.

## 6. Conclusions

Continued efforts must be carried out to better understand the potentially preventable factors associated with forgone medical care, such as cost. State and national policy makers are encouraged to seek ways to ameliorate the gaps in access to necessary medical care for the most vulnerable groups. Ongoing research should continue to incorporate geopolitical variations in policy that might affect access to necessary medical care.

## Figures and Tables

**Table 1:** Self-reported foregone care among adults, 2011–2015.

Variables		2011			2012			2013			2014			2015		
		Total	Age 18–64	65 and Older	Total	Age 18–64	65 and Older	Total	Age 18–64	65 and Older	Total	Age 18–64	65 and Older	Total	Age 18–64	65 and Older
		Percent	Percent	Percent	Percent	Percent	Percent	Percent	Percent	Percent	Percent	Percent	Percent	Percent	Percent	Percent
**Age**	18–64 years	19.50%			19.19%			18.53%			16.61%			15.29%		
	65 years and older	5.21%			4.94%			4.72%			4.91%			5.19%		
**Income**	Missing/don’t know	15.79%	19.90%	4.32%	14.91%	18.74%	4.71%	14.20%	17.83%	4.45%	13.39%	16.59%	4.64%	12.17%	15.00%	4.58%
	<$15,000	33.88%	38.48%	12.17%	33.85%	38.43%	11.09%	32.95%	37.11%	12.71%	29.06%	32.82%	11.58%	25.99%	29.13%	12.11%
	$15,000–<$25,000	28.58%	34.60%	7.54%	28.46%	34.35%	7.80%	27.78%	33.60%	7.18%	25.24%	30.41%	7.66%	23.22%	27.59%	8.52%
	$25,000–$35,000	21.08%	26.22%	4.26%	20.09%	24.96%	4.41%	19.58%	24.45%	4.26%	17.74%	22.17%	4.32%	16.89%	20.86%	4.98%
	$35,000–<$50,000	15.00%	17.91%	3.16%	14.47%	17.62%	2.85%	14.16%	17.29%	2.32%	12.66%	15.36%	3.46%	13.67%	16.71%	3.26%
	≥$50,000	6.23%	6.75%	1.91%	6.44%	7.08%	1.77%	5.97%	6.66%	1.35%	5.66%	6.29%	1.82%	6.13%	6.73%	2.47%
**Sex**	Male	15.51%	17.46%	4.95%	15.02%	17.03%	4.81%	14.10%	16.10%	4.36%	12.41%	14.06%	4.80%	11.98%	13.42%	5.42%
	Female	18.31%	21.55%	5.40%	17.97%	21.33%	5.05%	17.63%	20.94%	5.00%	16.07%	19.13%	4.99%	14.49%	17.15%	5.00%
**Education**	Did not graduate High School	27.16%	32.64%	8.97%	27.73%	33.21%	8.95%	27.09%	32.13%	9.52%	25.11%	29.61%	9.43%	22.90%	26.89%	10.14%
	Graduated High School	18.38%	21.85%	4.88%	17.65%	21.07%	4.69%	16.68%	20.04%	4.19%	15.16%	18.04%	4.61%	13.88%	16.36%	4.63%
	Attended College or Technical School	17.17%	19.42%	4.42%	16.50%	18.89%	4.26%	16.04%	18.48%	3.93%	13.95%	16.06%	4.36%	13.48%	15.55%	4.49%
	Graduated from College or Technical School	8.82%	9.80%	3.03%	8.82%	9.96%	2.78%	8.28%	9.38%	2.58%	7.54%	8.62%	2.65%	7.12%	7.98%	3.08%
**Race/Ethnicity**	Hispanic	26.73%	28.36%	10.75%	26.46%	28.24%	9.31%	25.73%	27.18%	10.92%	22.90%	24.19%	10.72%	21.14%	22.30%	10.40%
	Other	23.28%	25.14%	11.94%	21.44%	23.36%	8.24%	20.37%	22.22%	8.06%	17.46%	19.18%	6.71%	18.53%	19.99%	7.72%
	American Indian or Alaska Native	22.10%	23.83%	11.16%	22.10%	23.45%	12.34%	22.96%	25.16%	8.81%	17.55%	18.98%	10.40%	19.66%	21.58%	8.34%
	Asian	12.50%	13.11%	7.77%	13.76%	14.40%	8.79%	11.61%	12.20%	6.77%	10.77%	11.49%	5.67%	10.05%	10.18%	9.10%
	Black or African American	23.41%	25.47%	8.68%	22.29%	24.37%	8.71%	20.94%	23.34%	7.31%	18.90%	20.95%	7.78%	17.06%	18.39%	10.12%
	White	13.53%	16.11%	3.98%	12.97%	15.64%	3.77%	12.62%	15.29%	3.64%	11.42%	13.79%	3.88%	10.42%	12.54%	3.83%
**Rurality**	Urban	13.89%	16.37%	4.83%	12.78%	15.68%	4.42%	11.28%	14.33%	4.03%	10.51%	13.27%	4.18%	9.64%	12.27%	4.55%
	Rural	15.23%	18.78%	5.18%	13.75%	17.52%	4.94%	12.93%	17.11%	4.34%	12.16%	15.87%	4.78%	10.71%	14.00%	4.92%
**Census Region**	South	20.14%	23.33%	5.68%	19.25%	22.44%	5.39%	18.62%	21.76%	5.28%	16.61%	19.42%	5.24%	15.87%	18.44%	5.70%
	North	13.38%	15.33%	5.16%	13.54%	15.72%	4.76%	13.02%	15.19%	4.25%	12.40%	14.36%	4.89%	11.28%	12.81%	5.45%
	Midwest	14.47%	16.65%	4.76%	13.95%	16.22%	4.33%	13.84%	16.13%	4.31%	12.53%	14.56%	4.43%	11.19%	12.91%	4.54%
	Western/Pacific	16.85%	19.23%	4.50%	16.98%	19.50%	4.74%	15.74%	18.14%	4.37%	13.74%	15.80%	4.77%	12.47%	14.28%	4.62%
**Medicaid Expansion in 2014**	Not Expanded	18.53% *	21.47% *	5.41% *	17.77% *	20.77% *	4.86%	17.15% *	20.09% *	4.88%	15.61% *	18.32% *	4.82%	14.89% *	17.33% *	5.40%
	Expanded	15.33% *	17.54% *	4.84% *	15.35% *	17.70% *	4.93%	14.70% *	17.02% *	4.47%	13.03% *	14.98% *	4.97%	11.64% *	13.29% *	4.92%

* indicates significant (*p* < 0.01) difference across Medicaid Expansion status.

**Table 2:** Bivariate Analysis for Forgone Care among individuals including state Medicaid expansion.

Variables			2011			2012			2013			2014			2015		
			OR	99% Confidence Intervals		OR	99% Confidence Intervals		OR	99% Confidence Intervals		OR	99% Confidence Intervals		OR	99% Confidence Intervals	
**Medicaid Expansion in 2014**	Age 18–64	Not Expanded vs. Expanded	1.285 *	1.225	1.347	1.219 *	1.160	1.282	1.225 *	1.167	1.286	1.273 *	1.208	1.341	1.368 *	1.296	1.443
	Age 65 and older	Not Expanded vs. Expanded	1.124	0.991	1.274	0.985	0.836	1.161	1.097	0.958	1.255	0.967	0.839	1.115	1.101	0.943	1.287

Bolded text and * significantly different (alpha = 0.01); Domain analysis using proc surveylogistic in SAS 9.4 were conducted to assess sub-group analyses.

**Table 3:** Adjusted Analysis for Forgone Care among individuals aged 19–64 years of age.

Variables		2011			2012			2013			2014			2015		
		OR	99% Confidence Intervals		OR	99% Confidence Intervals		OR	99% Confidence Intervals		OR	99% Confidence Intervals		OR	99% Confidence Intervals	
**Income**	Missing/don’t know	2.705 *	2.412	3.035	2.586 *	2.236	2.992	2.647 *	2.312	3.031	2.276 *	1.967	2.633	1.916 *	1.629	2.252
	<$15,000 vs. ≥$50,000	6.845 *	6.115	7.661	6.783 *	5.884	7.818	7.255 *	6.305	8.348	6.146 *	5.259	7.182	4.836 *	4.030	5.803
	$15,000–<$25,000 vs. ≥$50,000	6.194 *	5.589	6.864	6.327 *	5.572	7.185	7.018 *	6.191	7.956	5.254 *	4.585	6.021	4.238 *	3.603	4.985
	$25,000–<$35,000 vs. ≥$50,000	4.316 *	3.843	4.846	3.972 *	3.433	4.595	4.382 *	3.802	5.05	3.937 *	3.340	4.641	3.167 *	2.630	3.813
	$35,000–<$50,000 vs. ≥$50,000	2.814 *	2.520	3.142	2.679 *	2.341	3.066	2.920 *	2.541	3.355	2.690 *	2.319	3.12	2.630 *	2.205	3.138
**Sex**	Male vs. Female	0.743 *	0.696	0.794	0.753 *	0.694	0.817	0.699 *	0.644	0.76	0.726 *	0.663	0.795	0.716 *	0.646	0.795
**Education**	Did not graduate High School vs. Graduated from College or Technical School	1.544 *	1.372	1.739	1.675 *	1.442	1.946	1.812 *	1.556	2.11	1.892 *	1.598	2.239	1.935 *	1.607	2.331
	Graduated High School vs. Graduated from College or Technical School	1.305 *	1.196	1.425	1.339 *	1.2	1.494	1.397 *	1.246	1.566	1.406 *	1.247	1.586	1.468 *	1.284	1.678
	Attended College or Technical School vs. Graduated from College or Technical School	1.422 *	1.306	1.550	1.426 *	1.28	1.588	1.494 *	1.346	1.659	1.431 *	1.272	1.609	1.645 *	1.446	1.872
**Race/Ethnicity**	Hispanic vs. White	1.217 *	1.094	1.355	1.308 *	1.146	1.493	1.223 *	1.064	1.406	1.174 *	1.008	1.367	1.413 *	1.195	1.672
	Other vs. White	1.457 *	1.201	1.768	1.405 *	1.126	1.754	1.393 *	1.088	1.784	1.275	0.990	1.642	1.597 *	1.193	2.136
	American Indian or Alaska Native vs. White	1.226	0.957	1.570	1.096	0.821	1.464	1.296	0.929	1.808	0.771	0.580	1.025	1.450 *	1.051	2.002
	Asian vs. White	1.214	0.963	1.529	1.149	0.813	1.623	1.178	0.84	1.652	1.052	0.704	1.573	0.971	0.680	1.387
	Black or African American vs. White	1.101 *	1.005	1.226	1.113	0.985	1.258	1.030	0.923	1.15	1.011	0.890	1.148	0.968	0.829	1.129
**Rurality**	Urban vs. Rural	1.024	0.955	1.097	1.051	0.969	1.14	1.006	0.929	1.089	1.007	0.916	1.108	1.049	0.934	1.177
**Census Region**	North vs. South	0.730 *	0.653	0.816	0.782 *	0.681	0.898	0.726 *	0.642	0.821	0.823 *	0.721	0.938	0.844 *	0.718	0.992
	Midwest vs. South	0.718 *	0.659	0.783	0.739 *	0.671	0.814	0.741 *	0.673	0.815	0.789 *	0.710	0.876	0.754 *	0.665	0.855
	Western/Pacific vs. South	0.849 *	0.775	0.929	0.882 *	0.789	0.985	0.810 *	0.715	0.918	0.834 *	0.732	0.951	0.749 *	0.644	0.872
**State Median Income**	State Median Income (continuous variable)	1.00	1.00	1.00	1.00	1.00	1.00	1.00	1.00	1.00	1.00	1.00	1.00	1.00	1.00	1.00

Bolded text and * significantly different (alpha = 0.01); Model adjusted for income, sex, education, race/ethnicity, rurality (urban or rural), census region (north, south, east, and west), and the state average median household income by year. Domain analysis using proc surveylogistic in SAS 9.4 were conducted to assess sub-group analyses.

**Table 4:** Adjusted Analysis for Forgone Care among individuals aged 19–64 years of age including state Medicaid expansion.

Variables		2011			2012			2013			2014			2015		
		OR	99% Confidence Intervals		OR	99% Confidence Intervals		OR	99% Confidence Intervals		OR	99% Confidence Intervals		OR	99% Confidence Intervals	
**Income**	Missing/don’t know	2.706 *	2.412	3.036	2.586 *	2.235	2.992	2.647 *	2.311	3.030	2.276 *	1.966	2.633	1.927 *	1.640	2.265
	<$15,000 vs. ≥$50,000	6.849 *	6.119	7.666	6.784 *	5.886	7.82	7.252 *	6.303	8.345	6.165 *	5.275	7.205	4.849 *	4.043	5.817
	$15,000–<$25,000 vs. ≥$50,000	6.192 *	5.588	6.862	6.328 *	5.572	7.185	7.019 *	6.191	7.957	5.259 *	4.589	6.027	4.246 *	3.610	4.992
	$25,000–<$35,000 vs. ≥$50,000	4.314 *	3.842	4.844	3.972 *	3.433	4.596	4.383 *	3.803	5.051	3.935 *	3.338	4.638	3.164 *	2.627	3.809
	$35,000–<$50,000 vs. ≥$50,000	2.813 *	2.519	3.141	2.679 *	2.340	3.066	2.921 *	2.541	3.357	2.688 *	2.317	3.118	2.624 *	2.200	3.131
**Sex**	Male vs. Female	0.743 *	0.696	0.794	0.753 *	0.694	0.817	0.699 *	0.644	0.760	0.727 *	0.664	0.795	0.716 *	0.646	0.795
**Education**	Did not graduate High School vs. Graduated from College or Technical School	1.545 *	1.372	1.740	1.675 *	1.442	1.946	1.811 *	1.556	2.109	1.895 *	1.601	2.243	1.937 *	1.609	2.332
	Graduated High School vs. Graduated from College or Technical School	1.305 *	1.195	1.425	1.339 *	1.200	1.494	1.397 *	1.246	1.566	1.407 *	1.248	1.587	1.469 *	1.285	1.679
	Attended College or Technical School vs. Graduated from College or Technical School	1.422 *	1.305	1.549	1.426 *	1.280	1.588	1.494 *	1.346	1.659	1.431 *	1.273	1.61	1.645 *	1.446	1.872
**Race/Ethnicity**	Hispanic vs. White	1.219 *	1.095	1.356	1.308 *	1.146	1.493	1.223 *	1.064	1.406	1.175 *	1.008	1.368	1.414 *	1.196	1.673
	Other vs. White	1.459 *	1.202	1.770	1.405 *	1.126	1.754	1.394 *	1.088	1.785	1.273	0.988	1.640	1.594 *	1.193	2.132
	American Indian or Alaska Native vs. White	1.222	0.954	1.565	1.095	0.820	1.463	1.298	0.930	1.810	0.767	0.577	1.019	1.430 *	1.036	1.974
	Asian vs. White	1.216	0.965	1.532	1.149	0.813	1.624	1.177	0.840	1.650	1.057	0.707	1.580	0.980	0.686	1.399
	Black or African American vs. White	1.111 *	1.006	1.227	1.114	0.986	1.258	1.030	0.923	1.149	1.011	0.890	1.148	0.969	0.831	1.131
**Rurality**	Urban vs. Rural	1.023	0.955	1.097	1.051	0.969	1.140	1.006	0.929	1.090	1.006	0.915	1.106	1.049	0.935	1.177
**Census Region**	North vs. South	0.745 *	0.669	0.831	0.786 *	0.691	0.894	0.718 *	0.637	0.808	0.852 *	0.751	0.968	0.897	0.756	1.065
	Midwest vs. South	0.731 *	0.673	0.794	0.742 *	0.678	0.813	0.733 *	0.670	0.803	0.812 *	0.733	0.898	0.793 *	0.701	0.896
	Western/Pacific vs. South	0.880 *	0.802	0.965	0.890 *	0.797	0.993	0.794 *	0.702	0.898	0.888	0.776	1.016	0.827 *	0.705	0.969
**State Median Income**	State Median Income (continuous variable)	1.00	1.00	1.00	1.00	1.00	1.00	1.00	1.00	1.00	1.00	1.00	1.00	1.00	1.00	1.00
**Medicaid Expansion in 2014**	Not Expanded vs. Expanded	1.059	0.986	1.139	1.015	0.936	1.100	0.968	0.893	1.049	1.109 *	1.019	1.208	1.177 *	1.052	1.318

Bolded text and * significantly different (alpha = 0.01); Model adjusted for income, sex, education, race/ethnicity, rurality (urban or rural), census region (north, south, east, and west), the state average median household income by year, and Medicaid Expansion. Domain analysis using proc surveylogistic in SAS 9.4 were conducted to assess sub-group analyses.

**Table 5:** Adjusted Analysis for Forgone Care among individuals aged 65 years of age and older.

Variables		2011			2012			2013			2014			2015		
		OR	99% Confidence Intervals		OR	99% Confidence Intervals		OR	99% Confidence Intervals		OR	95% Confidence Intervals		OR	99% Confidence Intervals	
**Income**	Missing/don’t know	1.917 *	1.458	2.521	2.487 *	1.640	3.771	2.576 *	1.949	3.403	2.167 *	1.527	3.075	1.440	0.925	2.244
	<$15,000 vs. ≥$50,000	4.761 *	3.566	6.357	5.058 *	3.176	8.054	6.961 *	5.055	9.585	4.307 *	2.942	6.304	3.527 *	2.121	5.865
	$15,000–<$25,000 vs. ≥$50,000	3.491 *	2.674	4.558	4.109 *	2.731	6.182	4.221 *	3.200	5.568	3.413 *	2.456	4.743	2.655 *	1.702	4.141
	$25,000–<$35,000 vs. ≥$50,000	2.034 *	1.500	2.759	2.490 *	1.539	4.027	2.931 *	2.110	4.072	1.990 *	1.395	2.839	1.716 *	1.069	2.754
	$35,000–<$50,000 vs. ≥$50,000	1.604 *	1.171	2.196	1.654 *	1.032	2.650	1.473 *	1.068	2.032	1.710 *	1.194	2.448	1.249	0.766	2.037
**Sex**	Male vs. Female	0.987	0.855	1.140	1.027	0.840	1.256	0.987	0.838	1.162	1.085	0.920	1.279	1.271 *	1.031	1.566
**Education**	Did not graduate High School vs. Graduated from College or Technical School	1.562 *	1.242	1.964	1.430 *	1.013	2.017	1.485 *	1.160	1.901	1.682 *	1.259	2.247	1.854 *	1.291	2.662
	Graduated High School vs. Graduated from College or Technical School	1.141	0.927	1.405	1.043	0.758	1.435	0.967	0.775	1.206	1.175	0.914	1.510	1.122	0.828	1.520
	Attended College or Technical School vs. Graduated from College or Technical School	1.211	0.989	1.484	1.158	0.828	1.620	1.079	0.854	1.363	1.132	0.896	1.430	1.250	0.902	1.733
**Race/Ethnicity**	Hispanic vs. White	1.924 *	1.463	2.531	1.862 *	1.288	2.693	2.173 *	1.586	2.978	1.933 *	1.300	2.876	1.790 *	1.177	2.723
	Other vs. White	2.945 *	1.970	4.402	1.980 *	1.241	3.159	2.142 *	1.472	3.115	1.350	0.887	2.054	2.173 *	1.131	4.174
	American Indian or Alaska Native vs. White	1.828 *	1.016	3.287	2.566 *	1.259	5.230	1.742 *	1.021	2.974	2.048 *	1.094	3.834	2.099 *	1.312	3.359
	Asian vs. White	2.925 *	1.546	5.536	3.363 *	1.365	8.285	1.656	0.827	3.316	1.199	0.544	2.643	4.252 *	1.460	12.384
	Black or African American vs. White	1.716 *	1.413	2.084	1.722 *	1.230	2.412	1.433 *	1.153	1.781	1.622 *	1.285	2.047	2.152 *	1.628	2.844
**Rurality**	Urban vs. Rural	0.964	0.839	1.107	0.927	0.800	1.073	0.971	0.841	1.120	0.909	0.785	1.052	0.966	0.800	1.168
**Census Region**	North vs. South	1.124	0.906	1.396	1.041	0.741	1.461	0.918	0.731	1.153	1.151	0.884	1.497	1.112	0.828	1.495
	Midwest vs. South	1.007	0.847	1.198	0.957	0.806	1.138	0.968	0.812	1.155	0.984	0.818	1.184	0.903	0.734	1.112
	Western/Pacific vs. South	0.849	0.688	1.047	0.991	0.778	1.264	0.817	0.630	1.060	0.933	0.730	1.194	0.865	0.617	1.212
**State Median Income**	State Median Income (continuous variable)	1.00	1.00	1.00	1.00	1.00	1.00	1.00	1.00	1.00	1.00	1.00	1.00	1.00	1.00	1.00

Bolded text and * significantly different (alpha = 0.01); Model adjusted for income, sex, education, race/ethnicity, rurality (urban or rural), census region (north, south, east, and west), and the state average median household income by year. Domain analysis using proc surveylogistic in SAS 9.4 were conducted to assess sub-group analyses.

**Table 6:** Adjusted Analysis for Forgone Care among individuals aged 65 years of age and older including state Medicaid expansion.

Variables		2011			2012			2013			2014			2015		
		OR	99% Confidence Intervals		OR	99% Confidence Intervals		OR	99% Confidence Intervals		OR	99% Confidence Intervals		OR	99% Confidence Intervals	
**Income**	Missing/don’t know	1.918 *	1.458	2.521	2.49 *	1.641	3.777	2.573 *	1.947	3.400	2.172 *	1.531	3.080	1.440	0.924	2.243
	<$15,000 vs. ≥$50,000	4.761 *	3.566	6.356	5.063 *	3.178	8.064	6.967 *	5.059	9.594	4.311 *	2.948	6.305	3.529 *	2.121	5.870
	$15,000–<$25,000 vs. ≥$50,000	3.496 *	2.679	4.562	4.120 *	2.738	6.200	4.226 *	3.204	5.575	3.420 *	2.463	4.749	2.657 *	1.703	4.145
	$25,000–<$35,000 vs. ≥$50,000	2.036 *	1.502	2.760	2.491 *	1.54	4.028	2.936 *	2.113	4.078	1.996 *	1.400	2.846	1.718 *	1.070	2.757
	$35,000–<$50,000 vs. ≥$50,000	1.605 *	1.172	2.197	1.657 *	1.033	2.656	1.474 *	1.069	2.033	1.712 *	1.196	2.450	1.250	0.766	2.039
**Sex**	Male vs. Female	0.987	0.855	1.140	1.027	0.84	1.256	0.987	0.838	1.162	1.085	0.921	1.280	1.271 *	1.031	1.566
**Education**	Did not graduate High School vs. Graduated from College or Technical School	1.56 *	1.240	1.962	1.422 *	1.007	2.008	1.483 *	1.159	1.899	1.676 *	1.255	2.239	1.853 *	1.291	2.661
	Graduated High School vs. Graduated from College or Technical School	1.141	0.927	1.406	1.042	0.757	1.435	0.967	0.775	1.206	1.174	0.914	1.509	1.122	0.828	1.52
	Attended College or Technical School vs. Graduated from College or Technical School	1.212	0.989	1.485	1.158	0.828	1.62	1.078	0.854	1.362	1.131	0.896	1.429	1.251	0.902	1.734
**Race/ethnicity**	Hispanic vs. White	1.925 *	1.464	2.532	1.865 *	1.29	2.696	2.172 *	1.586	2.975	1.929 *	1.299	2.866	1.789 *	1.176	2.722
	Other vs. White	2.938 *	1.964	4.395	1.987 *	1.245	3.17	2.14 *	1.472	3.112	1.355	0.890	2.062	2.174 *	1.132	4.176
	American Indian or Alaska Native vs. White	1.833 *	1.018	3.300	2.589 *	1.266	5.292	1.748 *	1.025	2.983	2.048 *	1.096	3.824	2.102 *	1.314	3.364
	Asian vs. White	2.925 *	1.545	5.537	3.337 *	1.352	8.236	1.648	0.822	3.303	1.188	0.538	2.626	4.249 *	1.458	12.381
	Black or African American vs. White	1.715 *	1.412	2.083	1.720 *	1.23	2.406	1.431 *	1.151	1.779	1.620 *	1.284	2.044	2.151 *	1.627	2.844
**Rurality**	Urban vs. Rural	0.963	0.838	1.107	0.928	0.802	1.075	0.971	0.841	1.121	0.912	0.788	1.057	0.967	0.800	1.168
**Census Region**	North vs. South	1.104	0.890	1.369	0.979	0.717	1.335	0.890	0.713	1.110	1.076	0.842	1.374	1.101	0.810	1.495
	Midwest vs. South	0.989	0.838	1.169	0.899	0.763	1.060	0.940	0.793	1.114	0.925	0.778	1.099	0.894	0.729	1.096
	Western/Pacific vs. South	0.819	0.660	1.017	0.877	0.69	1.116	0.772	0.597	0.998	0.825	0.641	1.061	0.848	0.596	1.208
**State Median Income**	State Median Income (continuous variable)	1.00	1.00	1.00	1.00	1.00	1.00	1.00	1.00	1.00	1.00	1.00	1.00	1.00	1.00	1.00
**Medicaid Expansion in 2014**	Not Expanded vs. Expanded	0.943	0.809	1.099	0.815 *	0.688	0.967	0.911	0.779	1.066	0.814 *	0.694	0.953	0.969	0.794	1.183

Bolded text and * significantly different (alpha = 0.01); Model adjusted for income, sex, education, race/ethnicity, rurality (urban or rural), census region (north, south, east, and west), the state average median household income by year, and Medicaid Expansion. Domain analysis using proc surveylogistic in SAS 9.4 were conducted to assess sub-group analyses.
